# Age Structure, Sexual Dimorphism, and Ontogenetic Melanism in a Captive Population of the Endangered Freshwater Turtle *Mauremys reevesii* (Gray 1831)

**DOI:** 10.1002/ece3.73565

**Published:** 2026-04-30

**Authors:** Hakyung Kang, Jaeyoung Seo, Ju Hee Bae, Kyo Soung Koo, Yikweon Jang

**Affiliations:** ^1^ Department of Life Sciences and Division of EcoScience Ewha Womans University Seoul South Korea; ^2^ Seoul Zoo Seoul Grand Park Gwacheon South Korea; ^3^ Korean Environmental Geography Institute Sejong South Korea

**Keywords:** captive breeding, Geoemydidae, ontogeny, population structure, sexual size dimorphism

## Abstract

The age structure of animal populations plays a pivotal role in understanding their demographic dynamics and reproductive potential. Although carapace growth ring analysis is widely used to estimate turtle ages, environmental variability often causes irregular growth patterns, which can compromise the accuracy of this method. Research based on accurately determined ages remains limited despite its importance, especially when addressing vulnerable populations. This study investigated body size distribution and melanism in a captive population of 131 endangered freshwater turtles, *Mauremys reevesii* , with precisely known ages. The mean ages were 9.4 ± 3.9 years (maximum 16) for females and 5.5 ± 1.8 years (max 12) for males, indicating greater longevity in females compared to males. Females were significantly larger than males, with slower growth but larger asymptotic size, demonstrating female‐biased sexual size dimorphism. Sex could be distinguished from three years of age onward. Melanism appeared only in males, first being observed at 4 years of age, and the progression followed the order of eyes, claws, skin, and shell. The stage of melanism in male *M. reevesii* was significantly influenced by age rather than body size. These findings provide the first detailed age‐based dataset on growth and melanism in this critically endangered species. This study offers a scientific basis for more accurate population assessments and the development of targeted conservation strategies, while helping to bridge the gap between wild and captive populations.

## Graphical abstract

Effectively illustrates the sexual size dimorphism in males and females of endangered *Mauremys reevesii* .

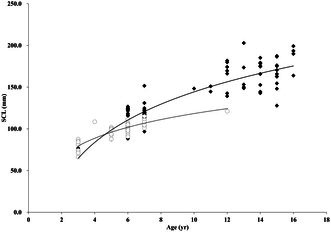

## 1 Introduction

A comprehensive understanding of population structure is essential for evaluating both historical and contemporary changes in species distribution and abundance, as well as short‐term demographic fluctuations (DeSante et al. 2001; Schowalter 2022). Such insights enable assessments of species vulnerability to environmental disturbances, provide indicators of ecological health and cohort dynamics, and inform the development of evidence‐based conservation strategies (Beddington 1974; Roden et al. 2013; Pongsanarm et al. 2025). Robust population data also allow for more accurate estimation of key demographic parameters, such as minimum viable population size and effective sex ratio, thereby facilitating more efficient planning and management of artificial breeding programs (Browne and Hecnar 2007; Armstrong and Seddon 2008; Pilcher et al. 2015). Among the parameters describing population structure, age composition is particularly informative for elucidating demographic processes, including variation in population size, growth rates, and overall demographic stability (Coulson et al. 2001; Hohn 2009; Pilcher et al. 2015; Hoy et al. 2020). For example, the age at sexual maturity serves as a key determinant of individual reproductive potential and strongly influences a population's intrinsic growth rate (Heppell et al. 2000; Oli and Dobson 2003; Hutchings et al. 2012). However, obtaining precise age estimates in wild populations remains a major methodological challenge, particularly for long‐lived species (Hohn 2009).

Various methods for age determination have been developed to reflect the specific biological traits of different animal groups. Common examples include the analysis of annual growth rings in mammalian teeth (Veiberg et al. 2020), incremental lines in amphibian phalanges (Sinsch 2015), and otolith layers in fish (Vitale et al. 2019). In turtles (Testudines), a widely adopted approach involves examining the relationship between carapace growth rings and body size (Cagle 1950; Sexton 1959; Bernstein et al. 2018). This method has proven effective for age estimation, especially in younger individuals (Sexton 1959; Germano 1988; Jung et al. 2016). Moreover, carapace ring analysis can provide insights not only into individual or population age, but also into age‐related size distributions and sex ratios in both captive and wild populations (Takenaka and Hasegawa 2001; Jung et al. 2016). However, the reliability of this technique remains debated (Wilson et al. 2003), as its accuracy can be influenced by species‐specific variations in growth, individual physiological differences, and environmental conditions experienced by wild populations (Cagle 1950; Brooks et al. 1997; Germano and Bury 2009; Skorczewski and Andersen 2021). Such factors introduce variability in growth patterns and thereby complicate precise age estimation.

Melanism, defined as the expression of a black phenotype resulting from the accumulation of dark pigments in the integument, has been documented across a wide range of animal taxa (Tucker et al. 1995; Majerus and Mundy 2003; Bury et al. 2020). This coloration has been observed in several chelonian species, including the globally invasive red‐eared slider ( *Trachemys scripta elegans* ) and the endangered Reeves' turtle ( *Mauremys reevesii* ) (Yabe 1994; Tucker et al. 1995). Notably, melanized individuals have been reported to reach sexual maturity and exhibit reproductive capacity, suggesting a potential association between melanism and ontogenetic development (Lovich et al. 1990; Tucker et al. 1995). However, it remains unclear whether the degree of melanism in turtles is mainly associated with chronological age, somatic growth, or a combination of both.

*Mauremys reevesii* is primarily distributed across the Korean Peninsula and China (Lovich et al. 2011). This freshwater turtle is currently classified as Endangered (EN A2bcd + 4bcd) on the IUCN Red List (Van Dijk 2011). Population declines have been largely driven by overexploitation for traditional medicine, extensive habitat degradation, and increasing environmental pollution (Lovich et al. 2011; Bu et al. 2022). Globally, *M. reevesii* has suffered severe demographic contractions approaching near‐extinction levels, which have led to its current conservation status (Van Dijk 2011). In South Korea, the species is additionally listed as Endangered Wildlife Class II and designated as Natural Monument No. 453, emphasizing its pressing conservation concern (National Institute of Biological Resources 2019). However, the lack of basic demographic information—such as reliable population size estimates and detailed assessments of population structure—remains a major constraint on effective management and recovery planning. This knowledge deficit impedes the development of evidence‐based conservation strategies and limits the capacity to accurately evaluate population viability.

In this study, we examined the population structure of a captive population of *M. reevesii* by quantifying the age, sex, body size, and progression of melanism. Our study is based on a captive colony in which all individuals have precisely known ages and were raised under controlled conditions, enabling the generation of reliable baseline growth data. Measurements obtained at known ages reduce the uncertainty associated with age estimation in wild populations and provide a robust foundation for analyses of morphometric traits and ontogenetic changes (Heppell et al. 2000; Hohn 2009; Glen et al. 2025). We therefore compared morphometric traits between sexes and evaluated the relative effects of age and body size on melanism development. These findings complement previous research, which has primarily focused on wild populations, by providing essential baseline data for understanding the species' morphological and developmental patterns.

## 2 Materials and Methods

### 2.1 Rearing of Turtles

Since 2004, Seoul Grand Park Zoo, located in the city of Gwacheon, Gyeonggi‐do, South Korea, has implemented conservation management strategies and developed *ex‐situ* breeding protocols to support the population augmentation of *M. reevesii* within its captive breeding program (Korea National Park Service 2012; Jo et al. 2019). The project started with individuals collected from the wild and donated by citizens, and by 2011, 19 female and 7 male *M. reevesii* were maintained at the breeding facility. The turtles were housed and bred under controlled conditions and fed a diet consisting primarily of loach (family Cobitidae) and commercially formulated turtle feed three times per week (Korea National Park Service 2012). Upon hatching, the year of emergence for each neonate was recorded, with each individual assigned a unique alphanumeric identifier to enable longitudinal monitoring and systematic data collection. This protocol allowed for comprehensive tracking of individual life histories within the population. Despite the established management protocols, reproductive success declined substantially from 2010 to 2013, resulting in few or no successful hatchlings during this period.

### 2.2 Data Collection

On June 28, 2020, we conducted a survey of the *M. reevesii* population at Seoul Grand Park Zoo, which included 92 females, 37 males, and 2 juveniles. For each individual, age, sex, morphometric parameters, and degree of melanism were recorded. Age determination was based on the zoo's birth and growth records dating back to 2005, ensuring accurate age information for individuals hatched within the captive breeding program. Individuals obtained through donation or rescue operations were excluded due to the lack of reliable age information. The sex of *M. reevesii* was determined by cloacal position, a recognized secondary sexual characteristic in chelonians. The female cloaca is situated nearer to the rear end of the plastron, whereas in males it extends beyond the posterior edge of the carapace and is accompanied by a longer and thicker tail (Lovich et al. 2011). Straight carapace length (SCL) and body weight (BW) were measured using digital calipers (Model 500‐193‐30, Mitutoyo, Japan) and an electronic scale (Model WK‐4CII, CAS, China), respectively.

**FIGURE 1:**
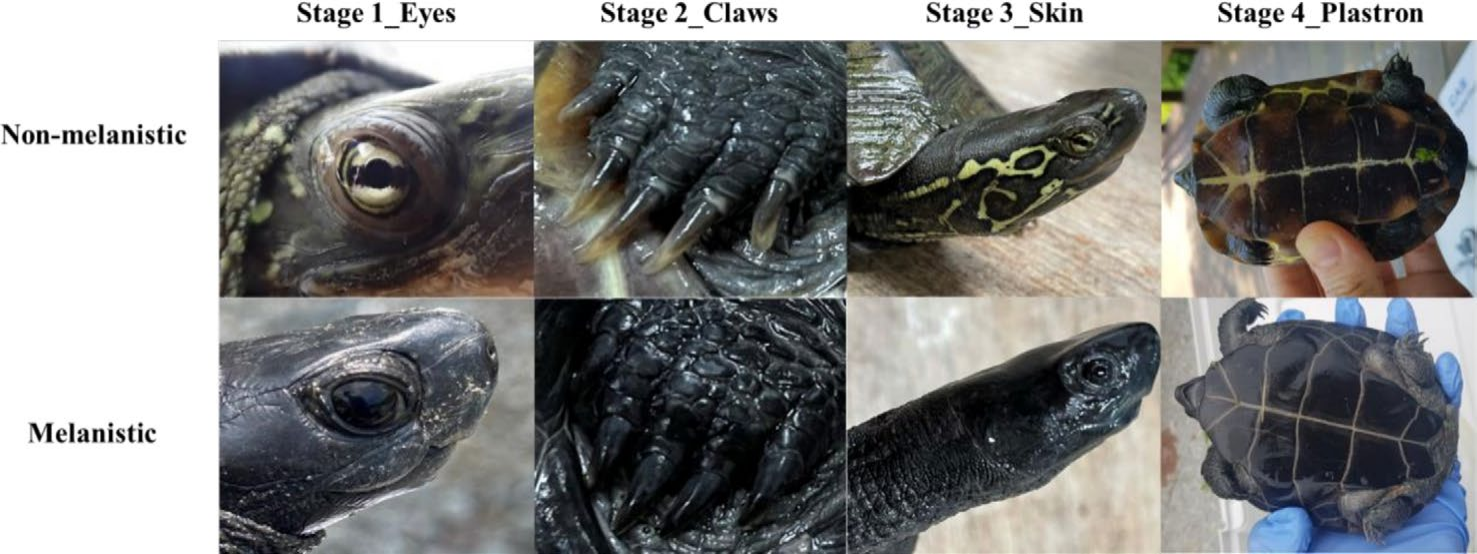
Stages of melanism in male *Mauremys reevesii* shown by progressive darkening of eyes, claws, skin, and plastron.

Melanization is characterized by distinct pigmentation changes observable across various body regions. In previous studies, melanism in *M. reevesii* has been classified into three developmental phases: (1) pre‐melanistic (non‐melanistic), (2) sub‐melanistic, and (3) melanistic (Yabe 1994). We observed that the development of melanism in *M. reevesii* does not occur simultaneously across all body regions. Therefore, we recorded the degree of melanism based on the pattern and progression of pigmentation changes in specific body parts. We categorized melanism into five stages: (0) non‐melanistic, (1) melanism in the eyes, (2) melanism in the eyes and claws, (3) melanism in the eyes, claws, and skin, and (4) melanism extending to the eyes, claws, skin, carapace, and plastron (Figure 1, Table 1). The detailed criteria for each stage are as follows: at stage 1, the light‐yellow iris darkens to black, rendering the pupil and iris indistinguishable; at stage 2, the fore and hind claws become black; at stage 3, the bright yellow patterns on the head and neck gradually fade as the skin darkens; and at stage 4, the brownish carapace and plastron become fully melanized and appear entirely black.

**TABLE 1:** Stages and characteristics of melanism occurring in male *Mauremys reevesii* .

Stage	Area of melanism	Description
0	None	No melanism (normal state)
1	Eyes	Eyes turn black
2	Eyes, claws	Eyes and claws turn black
3	Eyes, claws, skin	Skin gradually darkens and yellowish patterns fade
4	Eyes, claws, skin, carapace, plastron	Entire body including the shell completely turns black

This study was approved by the Seoul Zoo Scientific Research Committee (SZSRC 2020‐007). We adhered to the recommendations outlined in the ARRIVE guidelines, and all other methods were conducted in accordance with relevant guidelines and regulations set by the respective authorities.

### 2.3 Data Analysis

Homogeneity of variance in age and body size between sexes was assessed using Levene's test. As the assumption of homoscedasticity was violated, subsequent analyses employed nonparametric methods. Differences in age between sexes were analyzed using the Wilcoxon rank‐sum test.

Sexual size dimorphism was evaluated by comparing morphometric variables between sexes using Quade's nonparametric ANCOVA while controlling for the effect of age. Age‐specific growth in SCL was modeled using the von Bertalanffy growth function (VBGF): $L\left(t\right)=L_{\infty }\left(1-e^{-k\left(t-t_{0}\right)}\right)$, where $L_{\infty }$ represents the asymptotic SCL, *k* is the growth coefficient, and *t*_0_ is the theoretical age at zero length (Von Bertalanffy 1938). The model was fitted separately for females (*n* = 92, age range: 3–16 years) and males (*n* = 37, age range: 3–12 years) using nonlinear regression. Juveniles (*n* = 2) were excluded from model fitting due to the small sample size. Initial parameter values were set to $L_{\infty }$ = 250 mm for females and 130 mm for males, *k* = 0.3 years^−1^, and *t*_0_ = 0, based on values approximately 1.1–1.3 times the observed maximum carapace length of the species. Parameter bounds were constrained to biologically realistic ranges ($L_{\infty }$: 200–350 mm for females and 100–200 mm for males; *k* > 0.01 years^−1^; *t*_0_: −5 to +2 years), based on values reported in previous studies on the species (Yabe 1994; Kang et al. 2025). Model fit was evaluated using the coefficient of determination: *R* ^2^ = 1 − Σ(yᵢ − ŷᵢ)^2^/Σ(yᵢ − ȳ)^2^.

Because melanism stages progress sequentially and unidirectionally, ordinal regression was used to examine the effects of age and body size on melanism advancement. Potential multicollinearity among independent variables was evaluated using variance inflation factor (VIF) values; as all VIF values were below 7, no variables were excluded. Juveniles were omitted from the analyses due to indeterminate sex. All statistical analyses were conducted using SPSS Statistics ver. 26 (IBM, USA) with a significance level (α) set at 0.05.

## 3 Results

### 3.1 Age Structure

The mean age of females (*n* = 92) from Seoul Grand Park Zoo was 9.4 ± 3.9 years, which was significantly higher than that of males (*n* = 37; 5.5 ± 1.8 years) (Wilcoxon *W* = 1327.0, *Z* = −5.743, *p* < 0.001). The maximum recorded ages were 16 years for females and 12 years for males. Two juveniles (*n* = 2) were 1 and 2 years old, respectively, and sex became distinguishable at 3 years of age.

### 3.2 Body Size and Sexual Size Dimorphism

**FIGURE 2:**
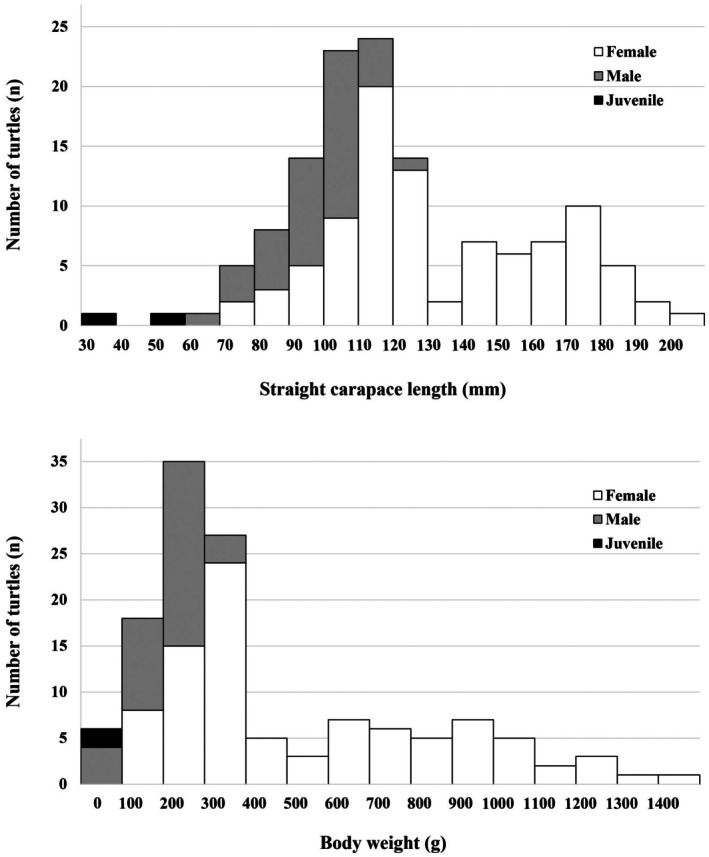
Distribution of straight carapace length (SCL, top) and body weight (BW, bottom) of *Mauremys reevesii* at Seoul Grand Park Zoo.

The mean SCL was 134.5 ± 3.3 mm for females (*n* = 92), 97.3 ± 2.1 mm for males (*n* = 37), and 47.4 ± 9.7 mm for juveniles (*n* = 2) (Figure 2, Table 2). In addition, the mean BW was 559.5 ± 35.2 g for females, 196.8 ± 10.7 g for males, and 16.7 ± 2.7 g for juveniles (Figure 2, Table 2).

**TABLE 2:** Descriptive statistics for straight carapace length (SCL) and body weight (BW) by sex.

Size parameter	Females (*n* = 92)	Males (*n* = 37)	Juveniles (*n* = 2)
SCL (mm)	134.5 ± 3.3 (73.6–202.9)	97.3 ± 2.1 (66.3–121.1)	47.4 ± 9.7
BW (g)	559.5 ± 35.2 (100.0–1,454.0)	196.8 ± 10.7 (68.0–333.0)	16.7 ± 2.7

*Note:* Values are presented as mean ± SD with the ranges (minimum–maximum) indicated in parentheses.

Sexual size dimorphism was evident in both SCL and BW. Quade's nonparametric ANCOVA revealed significant sex differences in SCL (*F*(1, 127) = 11.458, *p* = 0.001) and BW (*F*(1, 127) = 5.355, *p* = 0.022) after controlling for age, with females exhibiting larger values than males (mean ranks: SCL, females = 71.8, males = 48.1; BW, females = 69.8, males = 53.2).

### 3.3 Age‐Specific Growth Pattern

**FIGURE 3:**
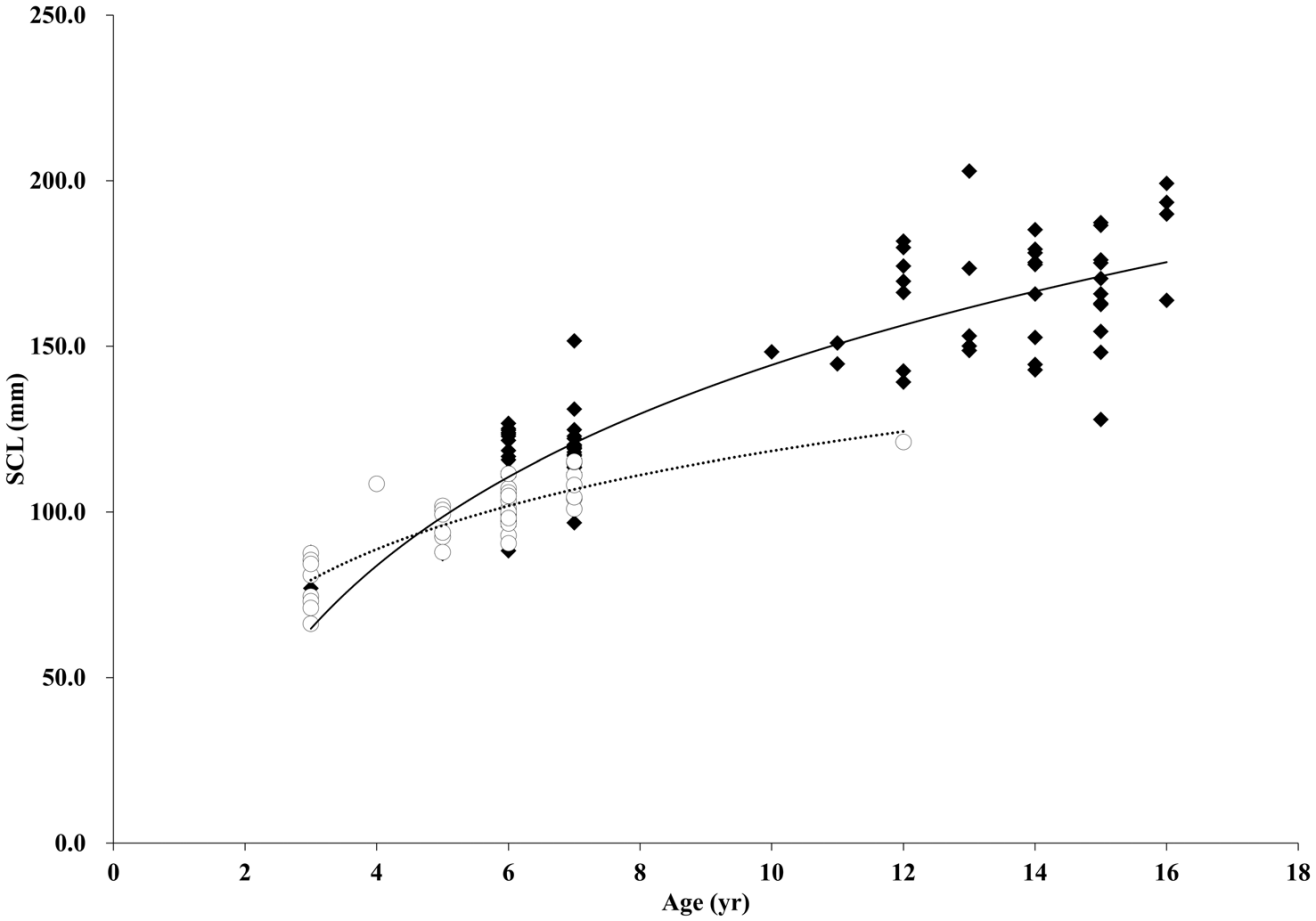
Relationship between age and straight carapace length (SCL) in captive *Mauremys reevesii* at Seoul Grand Park Zoo with fitted von Bertalanffy curves (females: filled diamonds with solid thick line; males: open circles with dashed line).

Growth in SCL, described using the von Bertalanffy growth function (VBGF) fitted separately for each sex, showed that females reached a larger asymptotic carapace length ($L_{\infty }$ = 227.3 ± 36.1 mm, *k* = 0.085 ± 0.036 years^−1^, *t*_0_ = −1.74 ± 1.60 years, *R* ^2^ = 0.813) than males ($L_{\infty }$ = 126.9 ± 13.8 mm, *k* = 0.221 ± 0.101 years^−1^, *t*_0_ = −1.43 ± 1.36 years, *R* ^2^ = 0.725). This indicates that females grow more slowly but attain a larger ultimate body size than males (Figure 3).

### 3.4 Melanism Development

**FIGURE 4:**
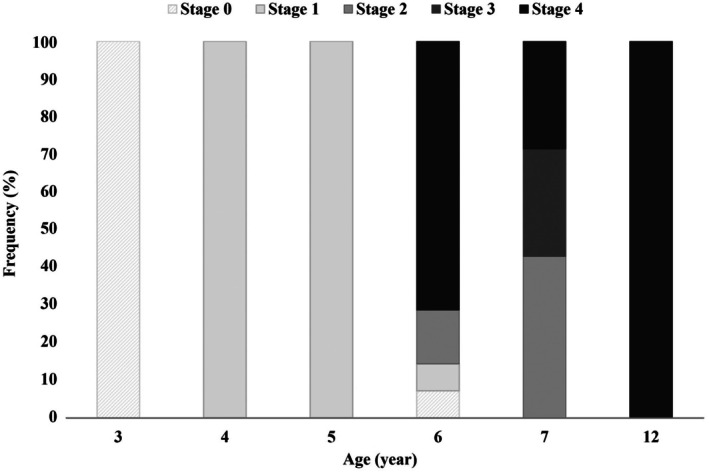
Frequency of proposed melanism stages in male *Mauremys reevesii* by age (Stage 0: none; Stage 1: eyes; Stage 2: eyes and claws; Stage 3: eyes, claws, and skin; Stage 4: eyes, claws, skin, carapace, and plastron).

Melanism was observed in 28 of 37 male individuals, whereas no melanism was detected in any females. The initial appearance of melanism was recorded in the fourth year post‐hatching (Figure 4). Accordingly, individuals aged 4 to 5 years exhibited stage 1 melanism, characterized by isolated ocular pigmentation, whereas from 6 years of age onwards, all stages (1 to 4) of melanism were observed (Figure 4).

The mean age of non‐melanized males was 3.3 years, with all individuals being 3 years old except for a single 6‐year‐old individual (Figure 4, Table 3). At stage 4, the mean age was 6.6 years, and individuals aged 6 years accounted for the highest proportion, representing 76.9% of the total. The oldest individual was 12 years old and exhibited stage 4 melanization. Stage 3 was observed only in two individuals, both aged 7 years. The advancement of melanism was significantly associated with age (*p* = 0.03), whereas body size variables (SCL and BW) had no significant effects (*p* > 0.05) (Table 4).

**TABLE 3:** The age, straight carapace length (SCL), and body weight (BW) according to the stages of melanism in male *Mauremys reevesii* .

Stage	N	Age (year)	SCL (mm)	BW (g)
0	9	3.3 ± 1.0 (3–6)	80.2 ± 9.9 (66.3–98.2)	114.4 ± 41.5 (68.0–204.0)
1	8	5.0 ± 0.5 (4–6)	99.0 ± 7.2 (87.9–108.5)	198.6 ± 44.2 (147.0–274.0)
2	5	6.6 ± 0.5 (6–7)	99.9 ± 8.4 (90.6–111.1)	210.2 ± 45.6 (152.0–253.0)
3	2	7.0 ± 0.0 (7)	109.8 ± 7.5 (104.5–115.1)	269.5 ± 48.8 (235.0–304.0)
4	13	6.6 ± 1.7 (6–12)	105.1 ± 7.3 (96.5–121.1)	236.3 ± 42.8 (191.0–333.0)

*Note:* Values are presented as mean ± SD with the ranges (minimum–maximum) indicated in parentheses.

**TABLE 4:** Ordinal regression analysis on the effects of age and body size on melanism in *Mauremys reevesii* .

Variables	Estimate	Std. error	Wald	df	Sig.	95% confidence interval	
						Lower bound	Upper bound
*Threshold*							
Stage = 1	11.908	9.429	1.595	1	0.207	−6.573	30.388
Stage = 2	13.045	9.486	1.891	1	0.169	−5.548	31.638
Stage = 3	13.372	9.505	1.979	1	0.159	−5.257	32.001
*Location*							
SCL	0.061	0.126	0.235	1	0.627	−0.186	0.308
BW	−0.009	0.022	0.165	1	0.684	−0.052	0.034
Age	1.441	0.664	4.701	1	0.030	0.138	2.743

## 4 Discussion

The captive population of *M. reevesii* examined in this study exhibited differences in developmental and melanistic stages compared to previously documented wild populations (Yabe 1994; Takenaka and Hasegawa 2001; Saka et al. 2014). Specifically, the onset of melanistic progression in male individuals was observed at 4 years post‐hatching in the captive population, in contrast to the 6‐year onset reported for wild populations (Yabe 1994). These discrepancies may be attributed to several factors. First, artificially optimized conditions in captivity may promote precocious sexual maturation and alter ontogenetic trajectories relative to wild individuals. Second, potential inaccuracies in age estimation methods might also lead to discrepancies between estimated and actual ages.

The divergent environmental conditions in captivity, compared to natural habitats, may induce precocious development and accelerate melanistic progression. *Ex‐situ* breeding facilities, including zoological institutions such as Seoul Grand Park Zoo, typically provide nutrient‐dense diets that exceed the nutritional quality of food resources available in the wild (Korea National Park Service 2012; Jayson et al. 2018; Hopkins III et al. 2022). Such enhanced nutritional provisioning may promote earlier ontogenetic progression, particularly with respect to sexual maturation (Swingle et al. 1993; Meers et al. 2016). In addition, elevated population densities and increased reproductive competition within captive environments may further influence developmental timing (Cureton II et al. 2010; Jones et al. 2019). Specifically, intense reproductive competition may drive the earlier onset of melanism in males, as melanism has been suggested to indicate reproductive capability (Cagle 1950). Such accelerated development may be associated with trade‐offs, including a reduction in final body size (Lovich et al. 1990; Ford and Seigel 1994; Bury et al. 2020). In summary, the distinctive nutritional and social conditions of captivity may collectively promote earlier sexual maturation and melanistic progression in *M. reevesii* , ultimately leading to differences in developmental rate, coloration, and morphology compared to wild populations. However, our data do not provide direct evidence for these mechanisms and should be tested in future studies.

In addition, discrepancies between actual and previously estimated ages at specific body sizes may result from methodological limitations in age estimation and variation in individual growth patterns. Unlike previous studies on wild populations that inferred age from counts of carapace and plastron annuli (Cagle 1950; Sexton 1959; Gibbons 1987; Bernstein et al. 2018), the present study examined individuals of known age. This difference in approach eliminates the uncertainty associated with indirect age inference. The use of growth rings for age estimation becomes increasingly unreliable in older individuals, as earlier growth lines may be obliterated (Bertolero et al. 2005; Jung et al. 2016). Moreover, both carapace annuli formation and subsequent growth rates are strongly influenced by environmental variability, such as delayed emergence from nests after hibernation (Kennett 1996; Berry 2002; Costanzo et al. 2008), leading to inconsistencies even among individuals within the same cohort.

The sex‐specific expression of melanism suggests that the trait is more likely driven by sexual selection than by natural selection favoring survival advantages (Lovich et al. 1990; Tucker et al. 1995). Previous studies have shown that melanistic males often display more aggressive courtship behavior and may gain reproductive benefits (Tucker et al. 1995; Thomas 2002). However, while some research indicates that melanism may not directly increase reproductive success, other studies propose it functions in social interactions (Cagle 1950; Lovich et al. 1990). For example, the reduced aggression between melanistic and non‐melanistic males suggests that melanism could serve as a recognition mechanism preventing misdirected intrasexual courtship attempts (Stone et al. 2015). Such behavioral patterns imply that melanism may act as a visual cue to distinguish reproductively experienced or ready males during social interactions, although this interpretation requires further testing. Although our study revealed a significant association between melanism stage and age in males, the specific factors that trigger its onset remain unidentified. Melanism is generally understood to be hormonally induced (Lovich et al. 1990; Roulin and Ducrest 2011); however, the precise regulatory mechanisms remain unclear. Therefore, further research involving a larger sample size of males is needed to elucidate the precise mechanisms underlying its development, including the potential influences of breeding experience and hormonal changes, and to assess whether melanism represents an adaptive trait.

## 5 Conclusion

This study presents a comprehensive assessment of the age structure, sexual dimorphism, and melanistic development in a captive population of the endangered freshwater turtle *M. reevesii* . The population exhibited a distinctly female‐biased age and size distribution, indicating greater longevity and larger body size in females compared to males. Such demographic patterns are expected to influence reproductive potential, cohort stability, and sex ratio management, emphasizing the importance of maintaining balanced demographic composition in captive breeding programs. The onset of melanism in males occurred earlier than in wild populations, implying that nutritional and environmental conditions in captivity may facilitate accelerated physiological development. The exclusive occurrence of melanism in males and its association with age, rather than body size, suggests that this trait may be primarily driven by sexual selection processes rather than somatic growth. Collectively, these findings expand our understanding of the species' ontogenetic and demographic characteristics under controlled environments. Integrating these morphological and developmental datasets into both *in‐situ* and *ex‐situ* management frameworks will contribute to more effective population monitoring, reinforcement, and recovery of this critically endangered turtle.

## Author Contributions

**Hakyung Kang:** conceptualization (equal), formal analysis (equal), funding acquisition (supporting), investigation (equal), methodology (equal), writing – original draft (equal), writing – review and editing (equal). **Jaeyoung Seo:** formal analysis (equal), writing – review and editing (equal). **Ju Hee Bae:** formal analysis (equal), investigation (equal), writing – review and editing (equal). **Kyo Soung Koo:** conceptualization (equal), formal analysis (equal), investigation (equal), methodology (equal), supervision (equal), writing – original draft (equal). **Yikweon Jang:** funding acquisition (lead), supervision (equal), writing – review and editing (equal).

## Funding

This work was supported by the Korea Environmental Industry & Technology Institute (KEITI), funded by the Ministry of Environment (grant number: RS‐2021‐KE001362). Also, Hakyung Kang was supported by the Basic Science Research Program through the National Research Foundation of Korea (NRF), funded by the Ministry of Education (grant number: RS‐2024‐00413107).

## Conflicts of Interest

The authors declare no conflicts of interest.

## Supporting information


**Data S1.** ece373565‐sup‐0001‐DataS1.xlsx.

## Data Availability

The relevant data supporting the findings of this study are included within this manuscript, and further inquiries can be directed to the corresponding author.
